# Supplementary material for: Botanical Control of Citrus Green Mold and Peach Brown Rot on Fruits Assays Using a Persicaria acuminata Phytochemically Characterized Extract
Source: Plants (Basel). 2021 Feb 24;10(3):425. doi: 10.3390/plants10030425 (PMC7996193; doi:10.3390/plants10030425)
Supplement: Supplementary file 1 [file plants-10-00425-s001.zip › plants-1074176-supplementary.pdf]

Supplementary material.

1. Compound descriptions including ^{1}H and ^{13}C NMR Spectra.

Compound **1**: MP: 48ºC. [α]D: -27º (*c* 1.00, $CHCl_3$). IR (KBr): 2927, 2850, 2726, 1722, 1680, 1642, cm^{-1}. ^{1}H NMR (300 MHz, $CDCl_3$): 9.48 (1H, *d*, *J*= 4.2 Hz, H-11); 9.42 (1H, *s*, H-12); 7.11 (1H, *m*, H-7); 2.78 (1H, *dddd*, *J*= 6.2, 2.1, 2.1, 2.1 Hz, H-9); 2.55-2.40 (1H, *m*, H-6α); 2.35-2.20 (1H, *m*, H-6β); 1.82 (1H, *m*, H-1β); 1.54-1.43 (3H, *m*, H-2α, 2β, 3β); 1.34 (1H, *td*, *J*= 4.0 and 13.4 Hz, H-1α); 1.26-1.16 (2H, *m*, H-3α, H-5); 0.92; (3H, *s*, Me-15); 0.91 and 0.89 (6H, 2*s*, Me-14 and Me-15). ^{13}C NMR (75 MHz, $CDCl_3$): 201.9 (HC=O); 193.2 (HC=O); 154.4 (=CH); 138.1 (=C); 60.2 (CH); 48,8 (CH); 41.7 (CH_2); 39.5 (CH_2); 36.8 (C); 33.0 (C); 33.0 (CH_3); 25.1 (CH_2); 21.9 (CH_3); 17.9 (CH_2); 15.2 (CH_3). MS (EI, 70 eV): m/z (%) = 234 [M^+], 216 [M^+ - H_2O], 206 [M^+ - CO], 191 [206 - Me].

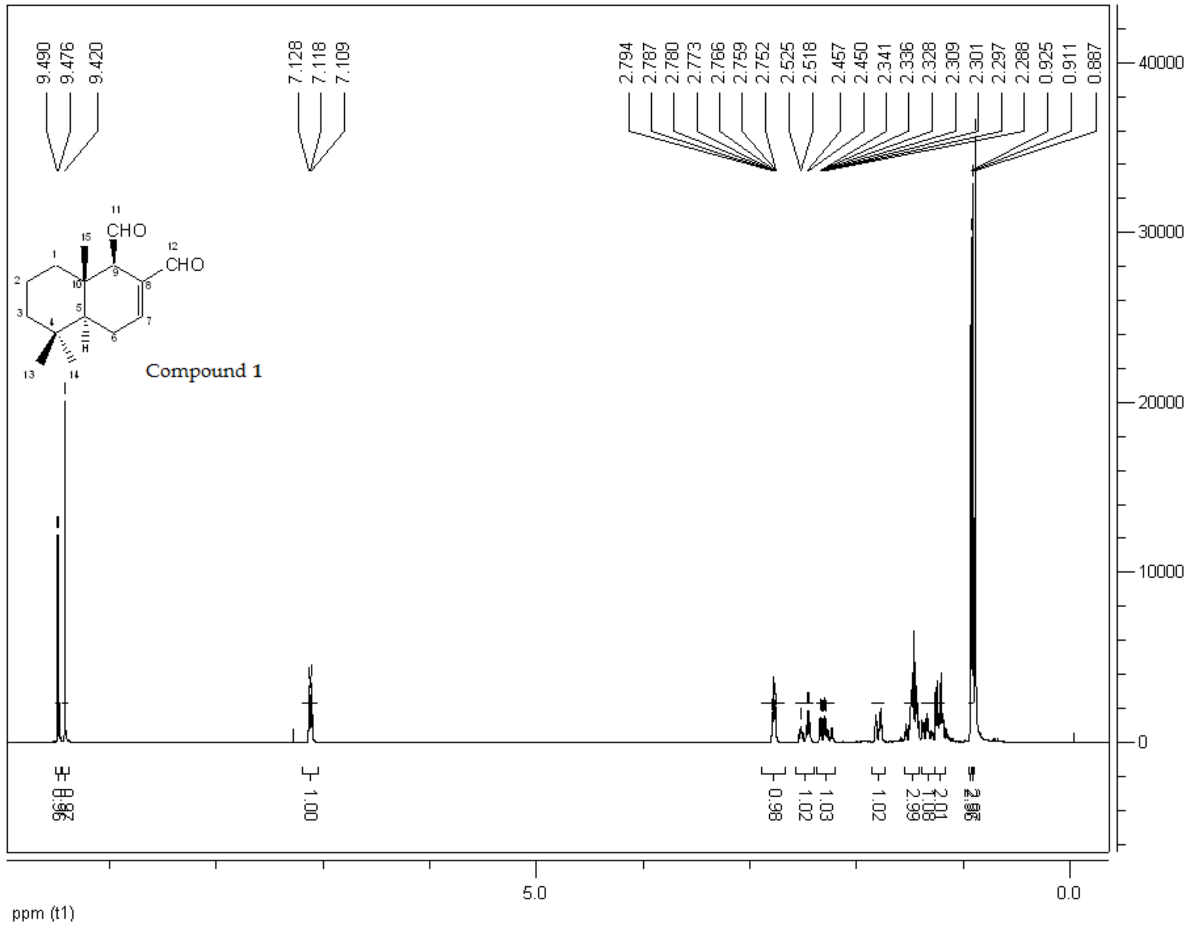

Figure S1: ^{1}H NMR spectra of polygodial (**1**).

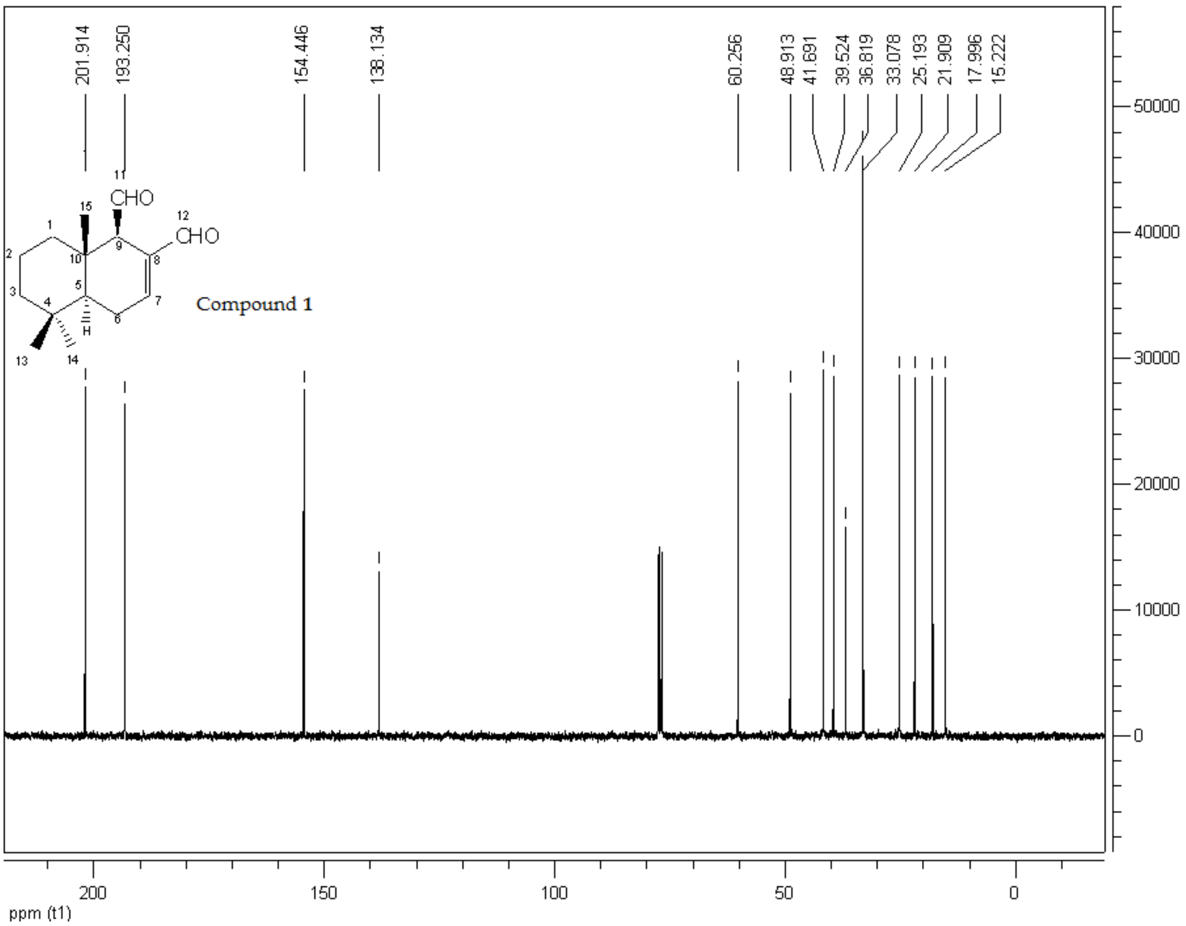

Figure S2: ^{13}C NMR spectra of polygodial (**1**).

Compound **2**: MP: 98ºC. $[\alpha]_D$: -15º (*c* 1.00, $CHCl_3$). IR (KBr): 3405, 2922, 1620 cm^{-1}. ^1H NMR (300 MHz, $CDCl_3$): 5.54 (1H, *m*, H-7); 3.85 (1H, *dd*, *J*= 3.3, 11.3 Hz, H-11B); 3.73 (1H, *dd*, *J*= 3.3, 11.3 Hz, H-11A); 1.97 (1H, *m*, H-6α); 1.95 (1H, *m*, H-1β); 1.92 (1H, *m*, H-6β); 1.86 (1H, *m*, H-9); 1.79 (3H, *s*, Me-12); 1.48 (2H, *m*, H-2α and 2β); 1.41 (1H, *m*, H-3β); 1.20 (2H, *m*, H-3α and H-5); 1.07 (1H, *m*, H-1α); 0.89, 0.87 and 0.85 (9H, 3*s*, Me-13, 14 and 15). ^{13}C NMR (75 MHz, $CDCl_3$): 132.9 (=C); 124.0 (=CH); 60.9 (H_2C-OH); 57.3 (CH); 49.9 (CH); 42.1 (CH_2); 39.8 (CH_2); 36.0 (C); 33.3 (CH_3); 32.9 (C); 23.6 (CH_2); 22.0 (CH_3); 21.9 (CH_3); 21.9 (CH_3); 18.8 (CH_2); 14.9 (CH_3). MS (EI, 70 eV): m/z (%) = 234 [M^+], 216 [M^+ - H_2O], 206 [M^+ - CO], 191 [206 - Me].

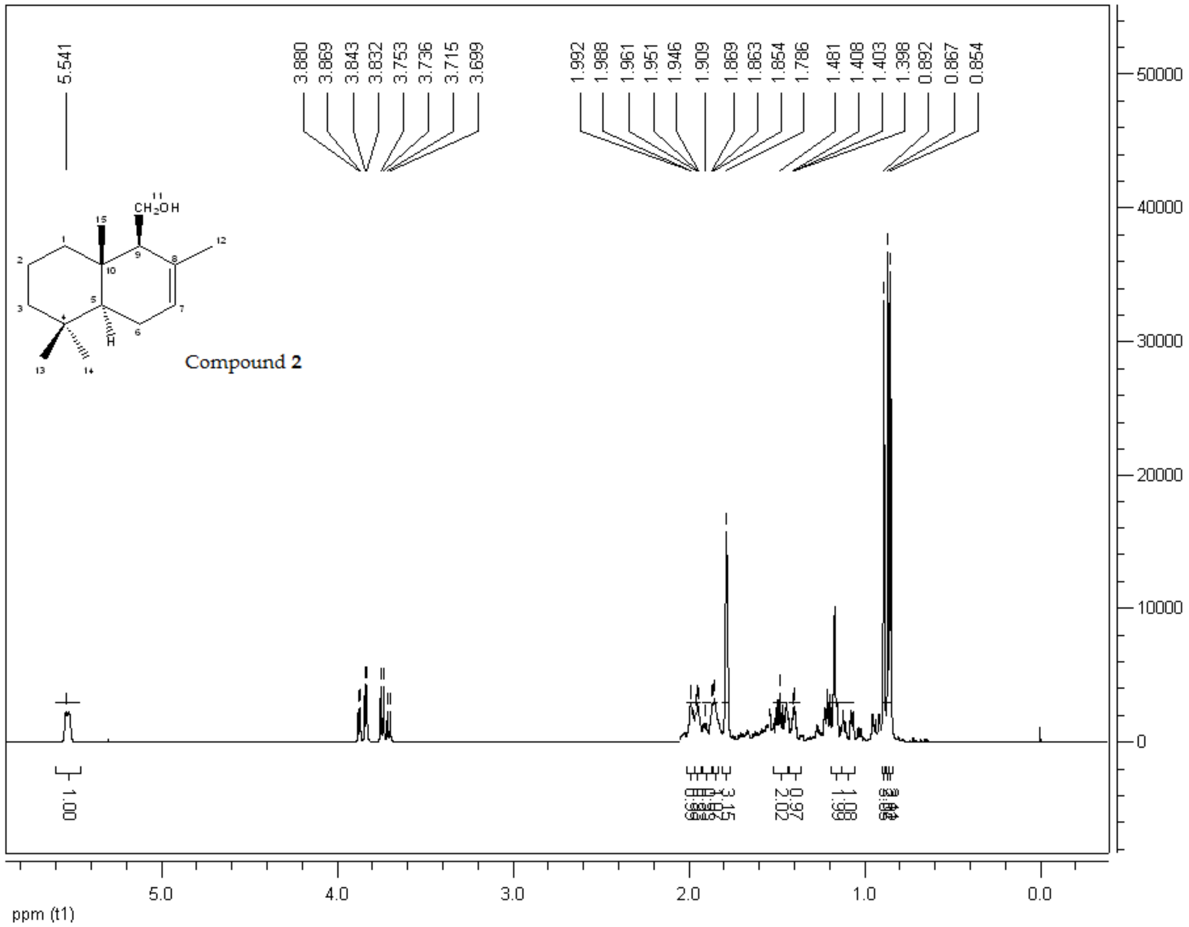

Figure S3: ^{1}H NMR spectra of drimenol (**2**).

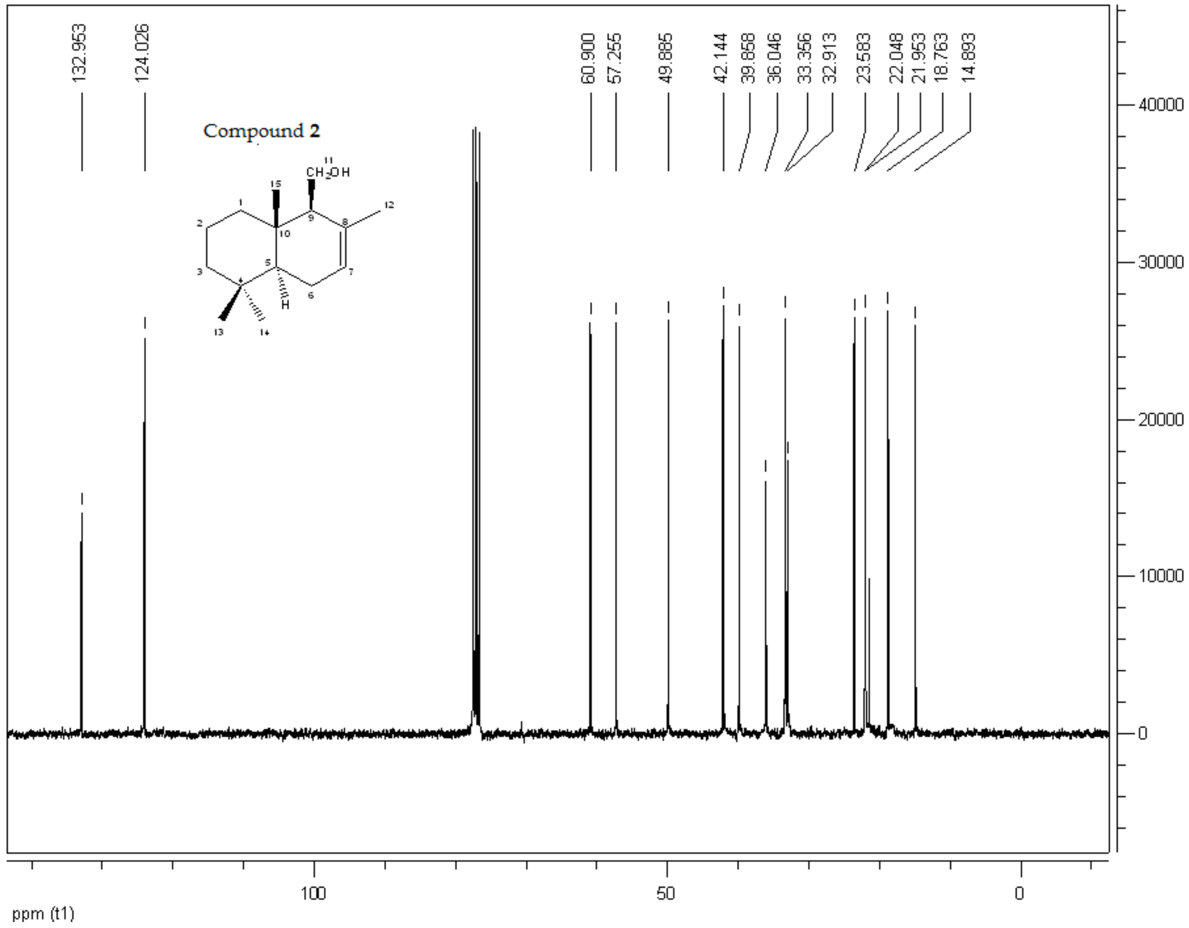

Figure S4: ^{13}C NMR spectra of drimenol (**2**).

Compound **3**: MP: 153ºC. $[\alpha]_D$: +70º (*c* 1.00, $CHCl_3$). IR (KBr): 1769, 1677 cm^{-1}. ^{1}H NMR (300 MHz, $CDCl_3$): 4.72 (1H, *ddd*, *J*= 2.8, 2.8, 16.9 Hz, H-1α); 4.62 (1H, *ddd*, *J*= 1.7, 3.5, 16.9 Hz, H-1β); 2.54-2.05 (2H, *m*, H-4α and β); 1.92-1.30 (9H, *m*, H-5α and β, H-5a, H-7α and β, H-8α and β, H-9α and β); 1.15 (3H, *s*, Me-9a); 0.93 and 0.89 (6H, 2*s*, Me-6α and β). ^{13}C NMR (75 MHz, $CDCl_3$): 174.5 (C=O); 170.8 (=C); 123.4 (=C); 68.2 (CH_2); 51.2 (CH); 41.6 (CH_2); 36.6 (CH_2); 36.0 (C); 33.2 (C); 33.2 (CH_3); 21.4 (CH_2); 21.4 (CH_3); 20.9 (CH_3); 18.3 (CH_2); 18.0 (CH_2). MS (EI, 70 eV): m/z (%) = 234 [M^+], 216 [M^+ - H_2O], 206 [M^+ - CO], 191 [206 - Me].

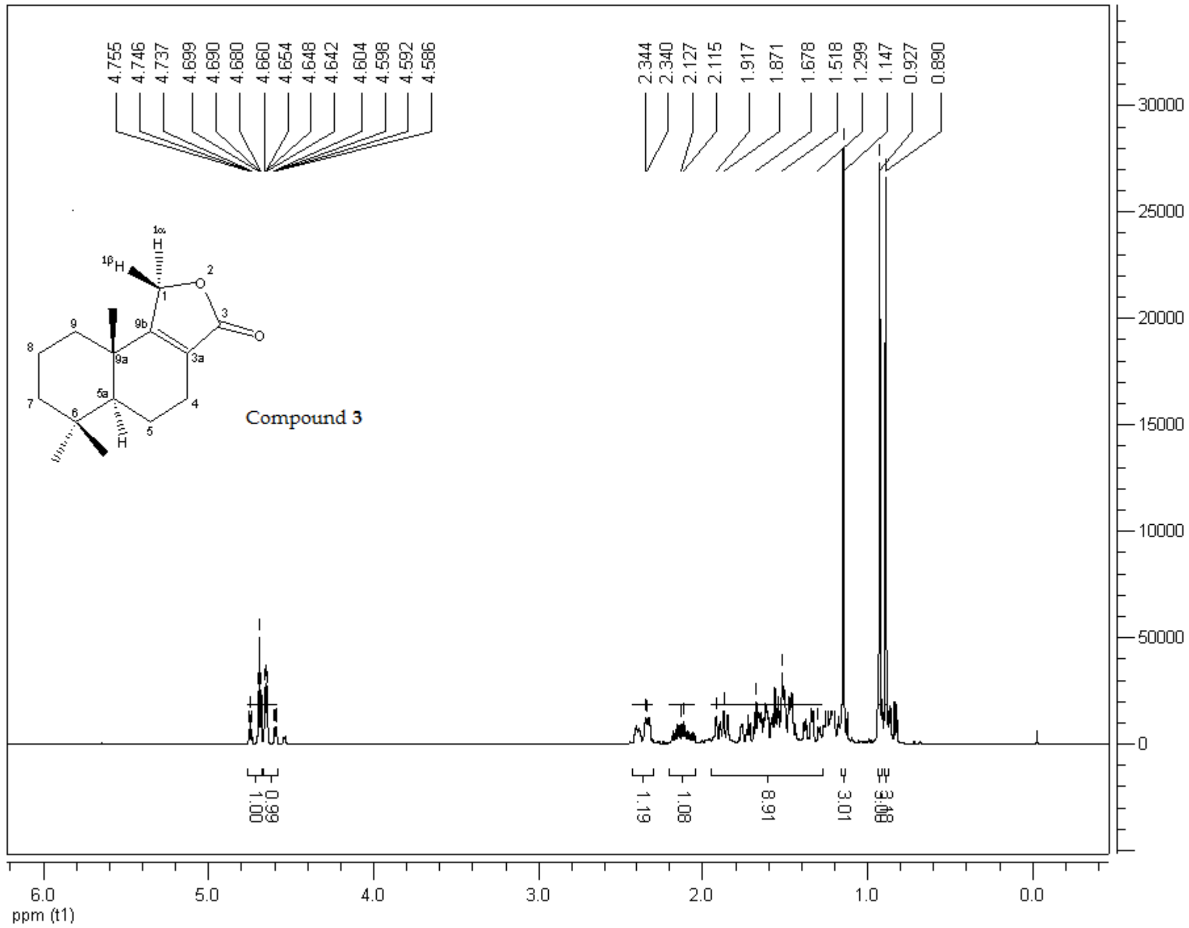

Figure S5: ^{1}H NMR spectra of confertifolin (**3**).

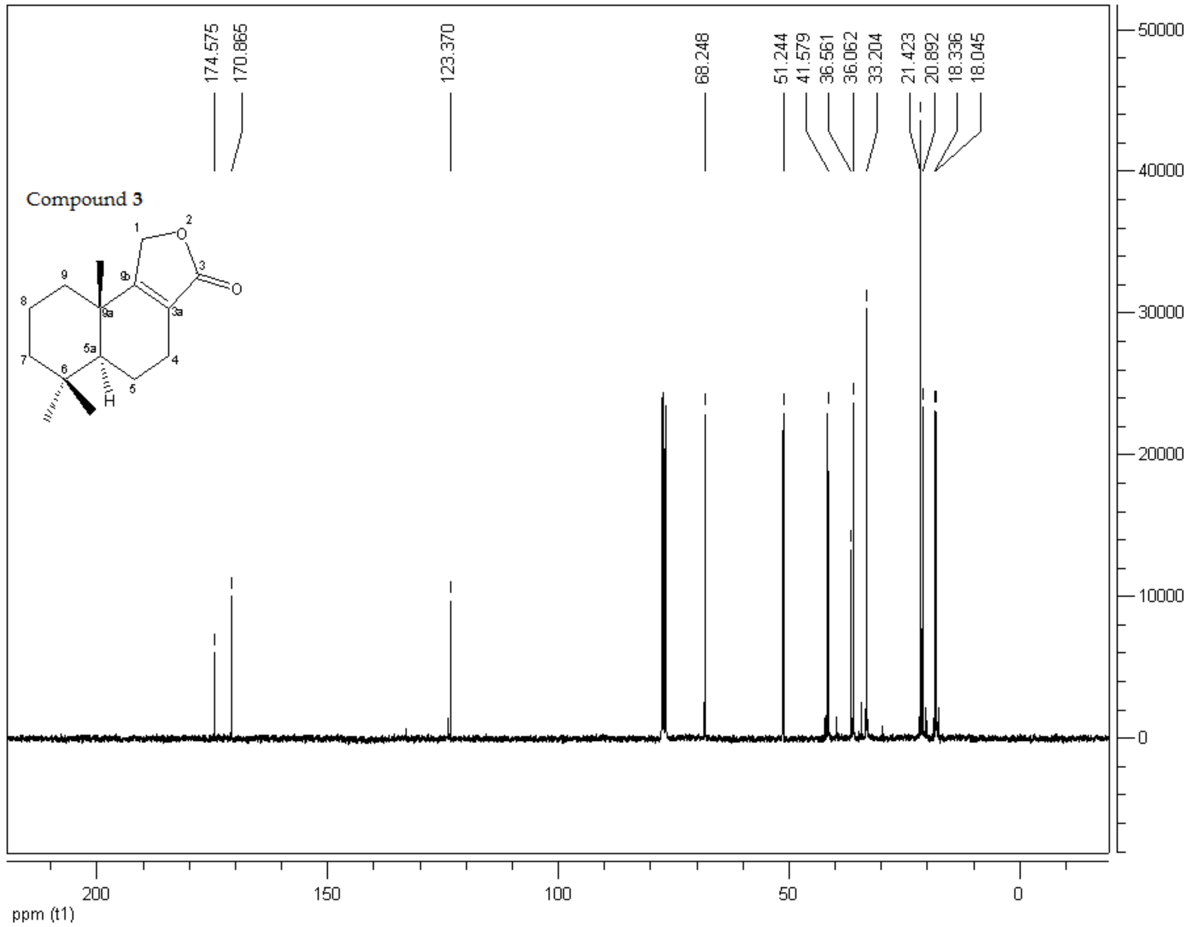

Figure S6: ^{13}C NMR spectra of confertifolin (**3**).

2. Gas chromatograms and Total Ion Chromatogram (TIC) areas obtained for different solution concentrations (0.5, 0.25 and 0.125 mg/mL) of pure polygodial and for EtOAc extract of *P. acuminata* (1 mg/mL). A mass spectrum is also showed.

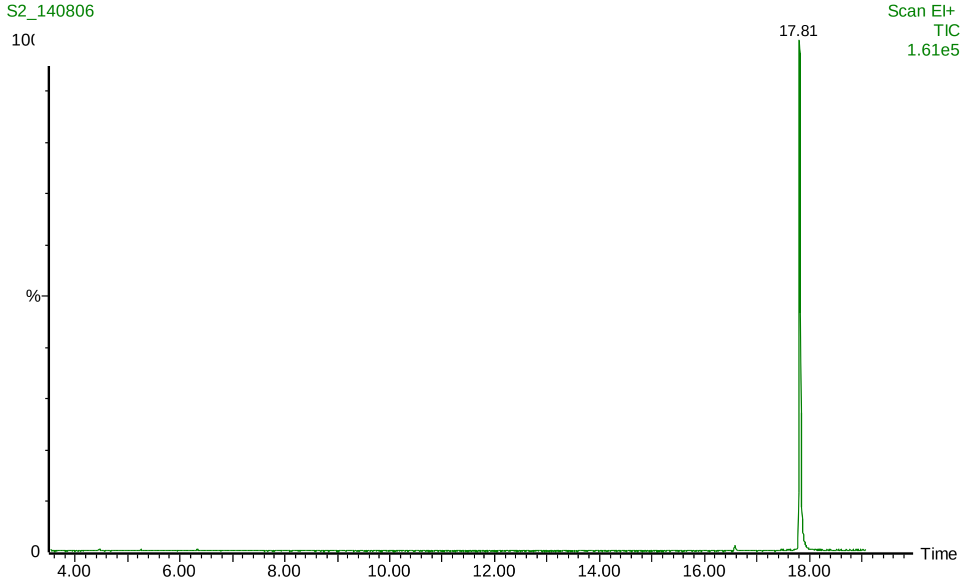

Figure S7: Gas chromatogram and Total Ion Chromatogram (TIC) area obtained for 0.5 mg/mL solution of polygodial (**1**).

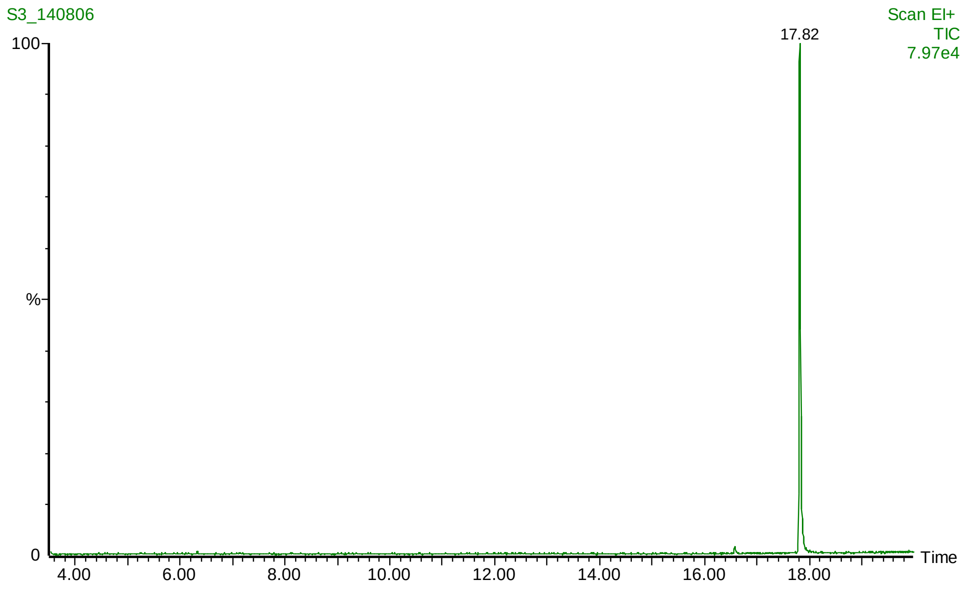

Figure S8: Gas chromatogram and Total Ion Chromatogram (TIC) area obtained for 0.25 mg/mL solution of polygodial (**1**).

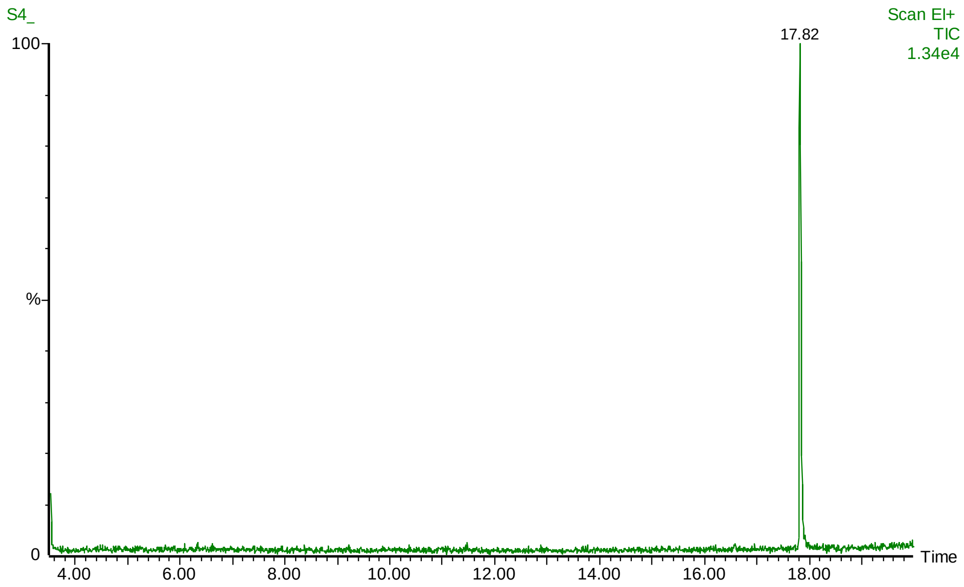

Figure S9: Gas chromatogram and Total Ion Chromatogram (TIC) area obtained for 0.125 mg/mL solution of polygodial (**1**).

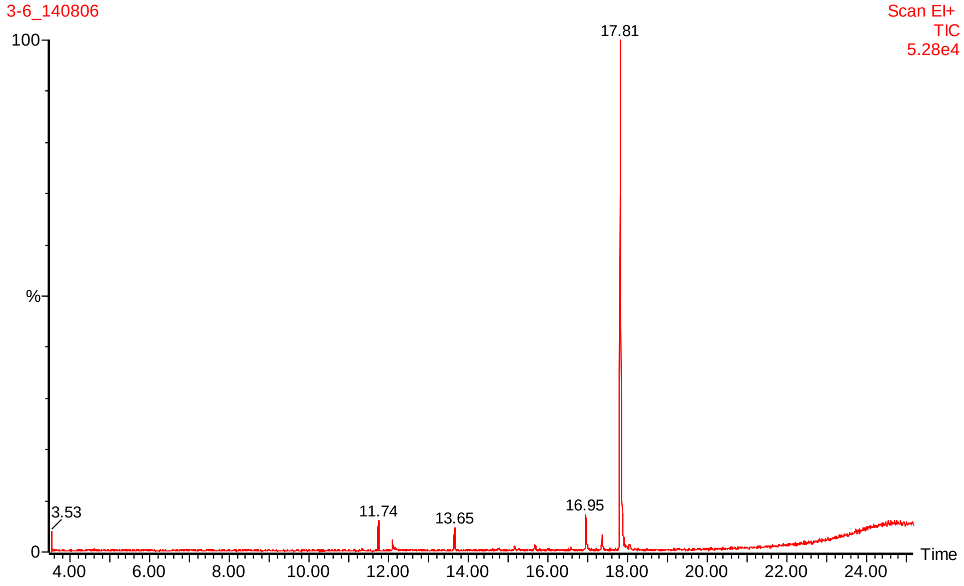

Figure S10: Gas chromatogram and Total Ion Chromatogram (TIC) area obtained for Ethyl Acetate extract of *P. acuminata* (1 mg/mL).

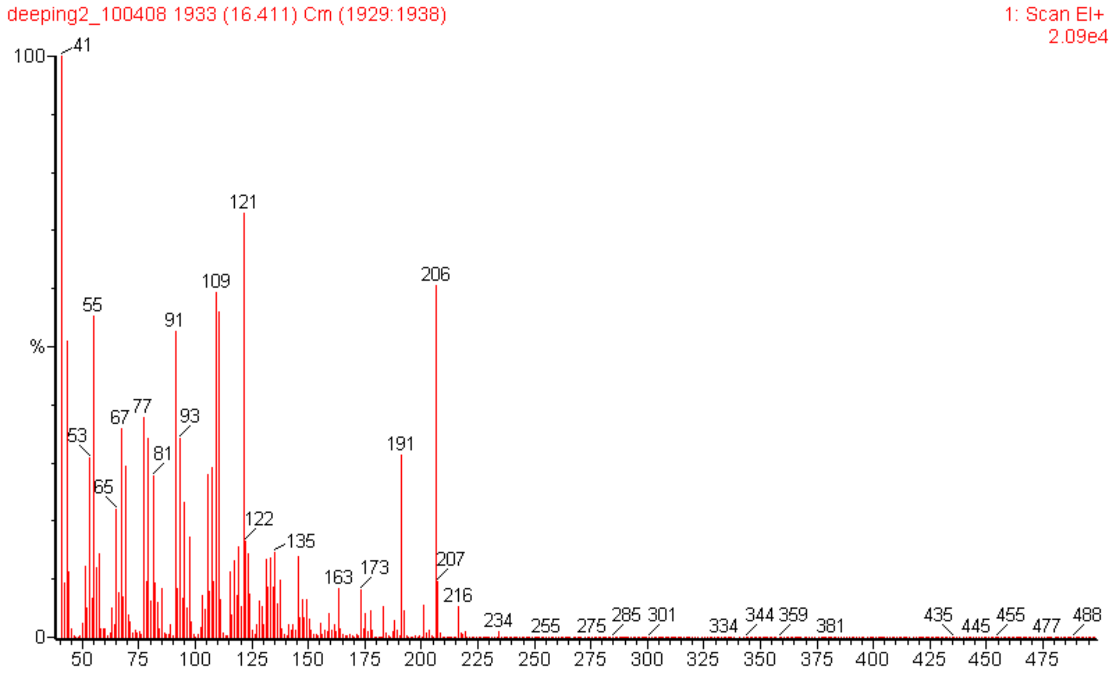

Figure S11: Mass spectrum of pure polygodial (**1**).
